# A Systematic Review of Building Fire Safety Practices in the UK: Approaches, Challenges and Recommendations

**DOI:** 10.1007/s10694-025-01801-2

**Published:** 2025-09-18

**Authors:** Jamiu A. Dauda, Muhammad A. Yamusa, Adekunle S. Oyegoke, Saheed O. Ajayi, Abdullahi B. Saka

**Affiliations:** 1 Construction Informatics and Digital Enterprise Laboratory, School of Built Environment, Engineering and Computing, Leeds Beckett University, Leeds, LS2 8AG UK; 2 Westminster Business School, University of Westminster, London, UK

**Keywords:** Fire, Fire safety practices, Fire safety training, Technological integration, Regulatory frameworks, Behavioural challenges

## Abstract

The catastrophic effect of fire incidents such as loss of lives, damage to building structures and economic loss, underscore the need for efficient fire safety in buildings, which has been a major subject of discussion in the UK. In this study, a comprehensive review of literature pertinent to building fire safety in the UK is presented. The study adopts systematic review approach, collected data from Scopus and analysed 51 qualified articles quantitively and qualitatively. The review shows a rise in publication since 2004, revealing prominent authors and keywords in building fire safety research. The review further identified the categories of fire safety practices in the UK, including technological innovations, mitigation, behavioural, and regulatory measures. Notable findings reveal the challenges in current practices including compliance and enforcement issues, maintenance of fire safety systems, public awareness and behavioural issues, technological adoption and integration challenges, and infrastructure and building design challenges. To address the challenges identified, proposed recommendations include fire safety training, simplifying and unifying regulations, maintenance and inspection of fire safety systems, fostering and upholding public trust, enhancing public awareness, integration of advanced technologies, and formulation of fire safety strategies. Additionally. the study further recommends more comparative research on international fire safety practices and social factors influence on fire regulations to effectively enhance fire safety practices in the UK.

## INTRODUCTION

The world, especially parts of Europe, including the United Kingdom (UK), is experiencing massive population growth due to urban immigration. The United Nations stresses that urban migration is expected to increase by 68% by the year 2055 [1]. This necessitates massive production of new buildings and retrofitting of existing buildings to cater for the teeming population growth to avoid unnecessary overcrowding that has significant potential for conflagration, fire ignition and spread within cities [2]. Meanwhile, retrofitting existing buildings and constructing new ones should be delivered sustainably to minimise greenhouse gas (GHG) emissions in line with the global climate emergency [3]. This often involves the use of sustainable materials such as timber, composites, lightweight concrete like sandcrete, and woodcrete for retrofit and new builds. This is owing to the benefits of these materials in terms of ease of replacement, less emissions, lightweight and biophilic benefits over other building materials like steel and concrete [4, 5]. However, the risk of fire is a major concern when it comes to using these sustainable materials, as many of them are highly combustible [4, 6].

Fire safety has become a major source of concern for building stakeholders. This is due to the devastating effect of fire incidents on properties, lives and well-being of people worldwide and in the UK [7, 8]. Abrassart et al. [2] submitted that over 180,000 people die in fires or from burn-related injuries worldwide every year. In the USA alone, an economic loss of around $15.9 billion was recorded in terms of property damage due to fire in 2021 [9]. Similarly, not less than 71 instant casualties were recorded from the catastrophic Grenfell Tower fire in 2017 in the UK [10]. This accentuates the critical need for effective strategies to prevent and manage fire incidents in buildings. As such, several efforts integrating regulatory, technological and behavioural dimension to fire safety have been made. Regulatory-oriented efforts include provisions in fire safety guidance documents such as Building Standards Technical Handbook: Domestic [11], Approved Document B (ADB) volume 1 [12], and BS 9991 [13]. Example of such regulatory provision is controlling smoke in the common corridors of multi-apartment residential structures in the event of fire [11]. Technological efforts include advancement in the development of automated assessment of fire risk [14], and system for automated code compliance checking of designs against fire safety regulations [15, 16]. These efforts enhance early detection of design non-compliances and thus mitigate fire risks in buildings. Additional efforts include the use of modern technologies like automated sprinkler systems to control fire spread [17]. Multiple studies have also explored the impacts of behaviour of building occupants in the event of a fire [8, 18]. With increasing calls for an overhaul of the building fire safety practices, a comprehensive review of studies relevant to fire safety in buildings is essential.

To address these issues, several studies have directed efforts towards ensuring fire safety in UK residential buildings to improve the regulatory guidance and optimise the fire safety process. For example, Kotzen et al. [19] analysed the associated fire safety risks and their impact on external living walls. Geoerg et al. [20] focused on evaluating the issues faced by pedestrians with reduced mobility in the event of fire. Hopkin & Fu [21] studied fire resistance periods for buildings. Although these efforts are crucial to fire safety, they are limited in their scope. The studies have failed to provide a comprehensive analysis of building fire safety practices to support the improvement of the existing building safety practices. As such, this paper presents a coordinated effort to holistically review the building fire safety practices in the UK, particularly residential buildings, to provide a deeper understanding of the existing building fire safety approaches. This stands to unearth issues, lessons and gaps in the existing literature on building fire safety approaches. Thereby, recommendations can be put forward to aid in policy development and bridging gaps. As a result, this research establishes a foundation for subsequent investigations that seek to examine building fire safety approaches within the UK through a comprehensive systematic review lens. The study, therefore, seeks to answer the research question: What are prevailing trends and main themes in building fire safety research in the UK?

This article is organised in five different sections with the first section offering an overview of the study’s context and articulating the aim of the review. The second section elaborates on the methodology adopted for the review including the criteria utilised for literature selection and the detailed analysis on the selected literature. The third section showcases the review’s findings, with subsections categorising the building fire safety approaches. Following this, the fourth section explores the lessons and gaps associated with building fire safety approaches. Finally, the last section concludes the paper and suggests opportunities for future research.

## Methodology

**Fig. 1:**
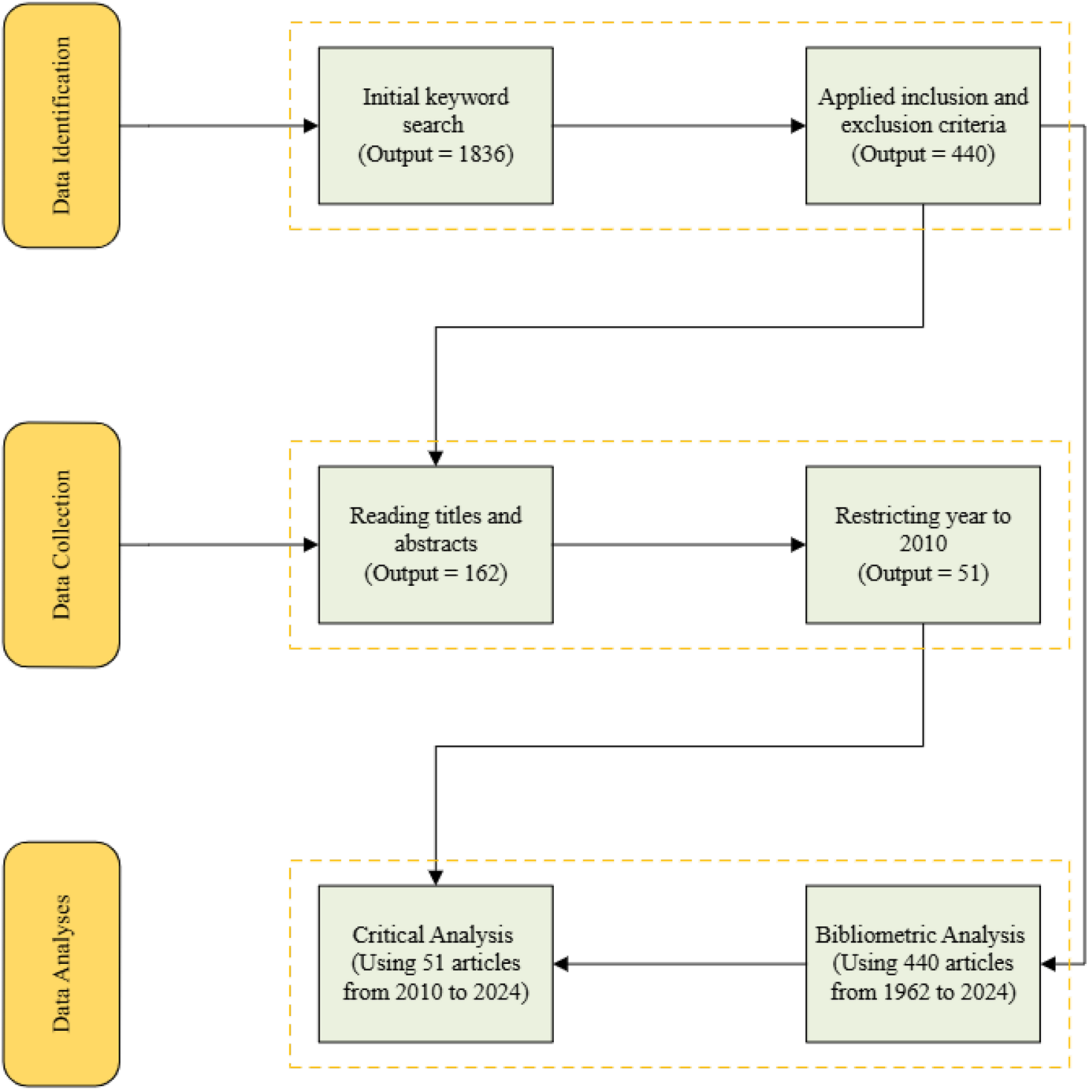
Research methodology

This study employed a systematic literature review (SLR) to analyse the state-of-the-art building fire safety practices in the UK. The SLR was conducted in three phases as shown in Fig. 1. The first phase is the data identification, which entails searching literature within the Scopus database. Scopus was selected because it is the largest comprehensive database for academic research output [22]. Limiting the search to Scopus allowed for focused literature identification while avoiding unnecessary duplication, as most of other sources like Web of Science, Google Scholar and ResearchGate often return overlapping but less comprehensive search results. In the second and third phases, identified relevant data was subsequently collected and analysed, respectively.

### Data Identification

This phase commenced with an initial search using a combination of keywords such as building, residential, construction, and fire safety practices to form appropriate search terms for the study. These terms were applied within the article title, abstract and keywords on Scopus database (i.e. TITLE-ABS-KEY), returning 1836 documents. After that, inclusion criteria such as building fire safety, studies related to fire safety practices in the UK, and within the Architecture, Engineering and Construction (AEC) industry were imposed. Subsequently, exclusion criteria were applied to remove non-English documents and non-journal articles to further restrict the search results to capture only significant literature. This was necessary as some grey literature and conference articles lacked sufficient details for systematic analysis. This led to the removal of 1396 articles, leaving 440 articles focused on building fire safety in the UK for the next phase. Finally, the search query used in this study is (TITLE-ABS-KEY (building AND residential* AND construction)) AND (fire AND safety AND practices) AND (LIMIT-TO (LANGUAGE, “English”)) AND (LIMIT-TO (SRCTYPE, “j”)) AND (LIMIT-TO (SRCTYPE, “j”)) AND (LIMIT-TO (COUNTRY, “UK”)) and was carried out in July 2024.

### Data Collection

In phase II, the 440 retained documents were collected in an Excel file and screened by two of the authors, who reviewed the titles and abstracts to identify the most relevant articles, resulting in 162 articles. This was then followed by a focus group discussion involving four of the authors, who agreed to impose additional criteria limiting article selections to publications from the year 2010. This was done to ensure that only recent efforts that are significant to the aim of the study are covered in this study. Most importantly, the year 2010 marks the period when the recommendations of the Regulatory Reform (Fire Safety) Order 2005 [23] were fully implemented across the UK. The FSO placed greater emphasis on risk assessments, preventative measures, and the responsibility of building owners to uphold fire safety standards. Additionally, the Building Regulations (2010) came into effect, replacing the Building Regulations 2000, and included Part B, specifically addressing fire safety. Therefore, 2010 represents a pivotal moment in the evolution of building safety practices, making it an ideal reference point for this study. At the end of the collection phase, the four authors agreed to retain 51 qualified articles for detailed review in the subsequent section. Summarily, Table 1 presents the details of search terms, exclusion and inclusion criteria.

**Table 1:** Details of search terms, exclusion and inclusion criteria

Category	Details
Search query	(building AND residential* AND construction) AND (fire AND safety AND practices)
Inclusion	1. Studies or articles from the UK 2. Architecture, Engineering and Construction (AEC) industry 3. Studies from 2010 for critical analysis
Exclusion	1. Exclude articles published in a language other than English language 2. Limit to only Journal articles

### Data Analysis

#### Bibliometric Analysis

The bibliometric analysis was conducted on all 440 retained articles prior to the limitation of the year 2010 to select qualified articles for critical analysis. The 440 articles were analysed to assess annual publication trends, publication sources, interactions among authors, and the co-occurrence of keywords. This analysis was conducted by constructing networks of author citations and keyword co-occurrence and generating visual maps from these networks to derive insights. This facilitates the visualisation and identification of trends, patterns, and developments within the research domain [25]. The analysis was conducted using VOSviewer, chosen for its advanced capabilities, open-source accessibility, and ease of use [25].

#### Critical Analysis

Subsequently, the outcomes from the data collection phase, which yielded 51 articles, were then critically reviewed and analysed to establish the approaches, best practices and limitations in the current building fire safety practices to come up with recommendations for improvement within the UK. The critical analysis was used to analyse the fire safety practices in the UK within the period set by the study. The critical analysis was used to address the last two research questions of the study. Prevailing discourse within the literature was reviewed using in-depth analysis. This reveals the main themes, along with the limitations and recommendations for improving the efficiency of fire safety practices research in the UK.

## Findings and Discussion

### Bibliometric Analysis Findings

#### Publication Trend

**Fig. 2:**
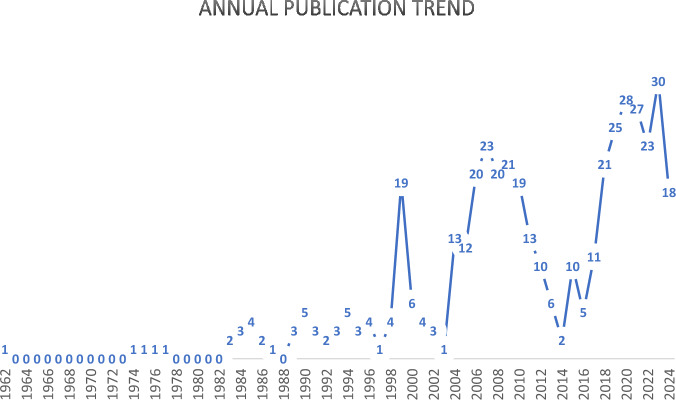
Annual publication trend

The analysis of the publication trend in the dataset reveals that the first publication on building fire safety practices in the UK was published in 1962. However, there were limited number of publications in the first four decades since the first publication, from 1962 to 2003 (Fig. 2). Only the year 1999 had 19 publications, while the highest recorded publications in any other year were five. Since 2004, publications have been increasing, averaging around 15 publications annually. The year 2023 recorded 30 publications, the highest total number of publications in a year while there are 18 publications in the year 2024 as at time of search. It is important to mention that data for 2024 is available only up to July, indicating the potential for further publications by the end of the year. The upward trend in publications since 2004 can be linked to developments in fire safety within the UK. For example, the implementation of the recommendations outlined in the Regulatory Reform (Fire Safety) Order 2005 (FSO, 2005) throughout the United Kingdom, the introduction of the Building Regulations 2010, and more recently, the introduction of the Building Safety Act (2022) which came about as a result of the disastrous Grenfell Fire of 2017.

#### Prominent Sources

Research sources serve an essential function in promoting the dissemination of knowledge among academics, fostering constructive critique, broadening conceptual frameworks, and presenting innovative theories. This examination is advantageous for readers, as it enables them to effectively pinpoint pertinent sources for their research, while simultaneously assisting authors in choosing suitable journals for manuscript submission [25]. The findings indicate that Fire Safety Journal, Fire Risk Management, Fire Prevention and Fire Engineers Journals, Fire Technology, and Building Engineer, with respective numbers of documents of 46, 39, 36, 35 and 24, are the foremost research sources for building fire safety. The top 10 sources (Table 2) contain 230 documents, making up 52% of the total documents. This indicates that the top 10 sources are the prominent sources for fire safety publications.

**Table 2:** Prominent research sources

S/N	Source title	Number of documents
1	Fire Safety Journal	46
2	Fire Risk Management	39
3	Fire Prevention and Fire Engineers Journals	36
4	Fire Technology	35
5	Building Engineer	24
6	Safety Science	15
7	Fire and Materials	14
8	Fire Engineers Journal	7
9	Journal of Building Engineering	7
10	Structural Engineer	7

#### Prominent Authors

**Fig. 3:**
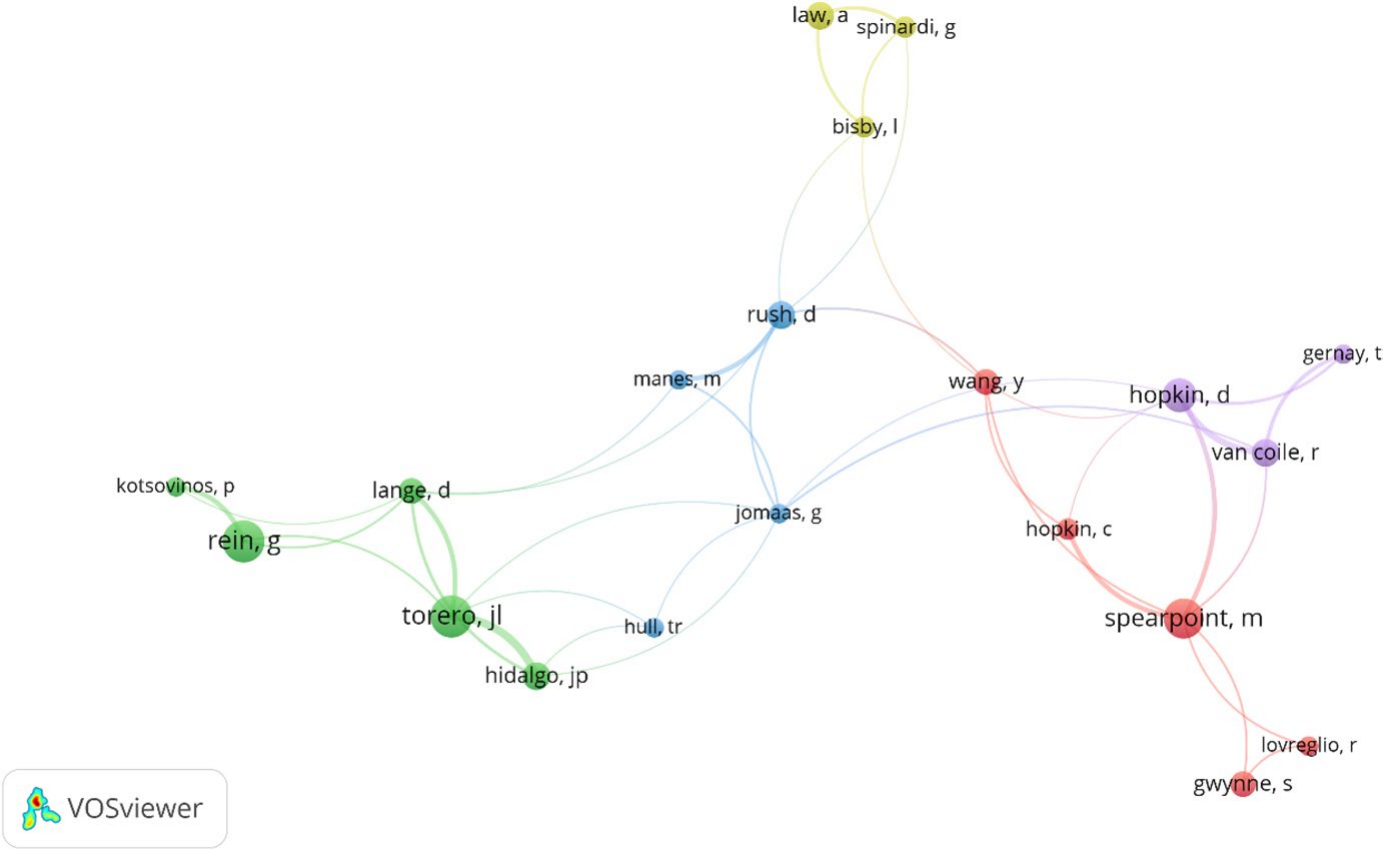
Prominent authors

To establish the prominent authors in building fire safety, a co-occurrence network was used to illustrate the collaborative relationships among authors. The collaboration among researchers from various institutions significantly enhances the exchange of knowledge and expertise [26]. This analysis not only highlights the leading authors but also aids in securing substantial collaborations for research projects. The top ten authors were identified and presented in Table 3. To determine the leading authors, criteria were established, requiring a minimum of five documents and five citations, and only 24 out of the 926 authors met these criteria. The analysis of total link strength revealed that Shields, T.J., Silcock, G.W.H., Boyce, K.E., Hopkin, D. and Van Coile, R. emerged as the five foremost authors in building fire safety research. This finding suggests that these authors have collaborated with other prominent researchers to produce significant contributions to building fire safety within the UK. This analysis offers valuable insights for scholars regarding the prominent and experienced researchers in building fire safety in the UK, who may be potential candidates for future collaborations and partnerships. (Figure 3)

**Table 3:** Prominent research authors

S/N	Author	Documents	Citations	Total link strength
1	Shields, T.J	16	427	23
2	Silcock, G.W.H	10	249	20
3	Boyce, K.E	10	353	18
4	Hopkin, D	13	153	16
5	Van Coile, R	8	185	13
6	Spearpoint, M	17	248	12
7	Hidalgo, J.P	7	165	10
8	Lange, D	7	62	10
9	Rein, G	18	584	9
10	Bisby, L	6	52	7

#### Keywords Analysis

**Fig. 4:**
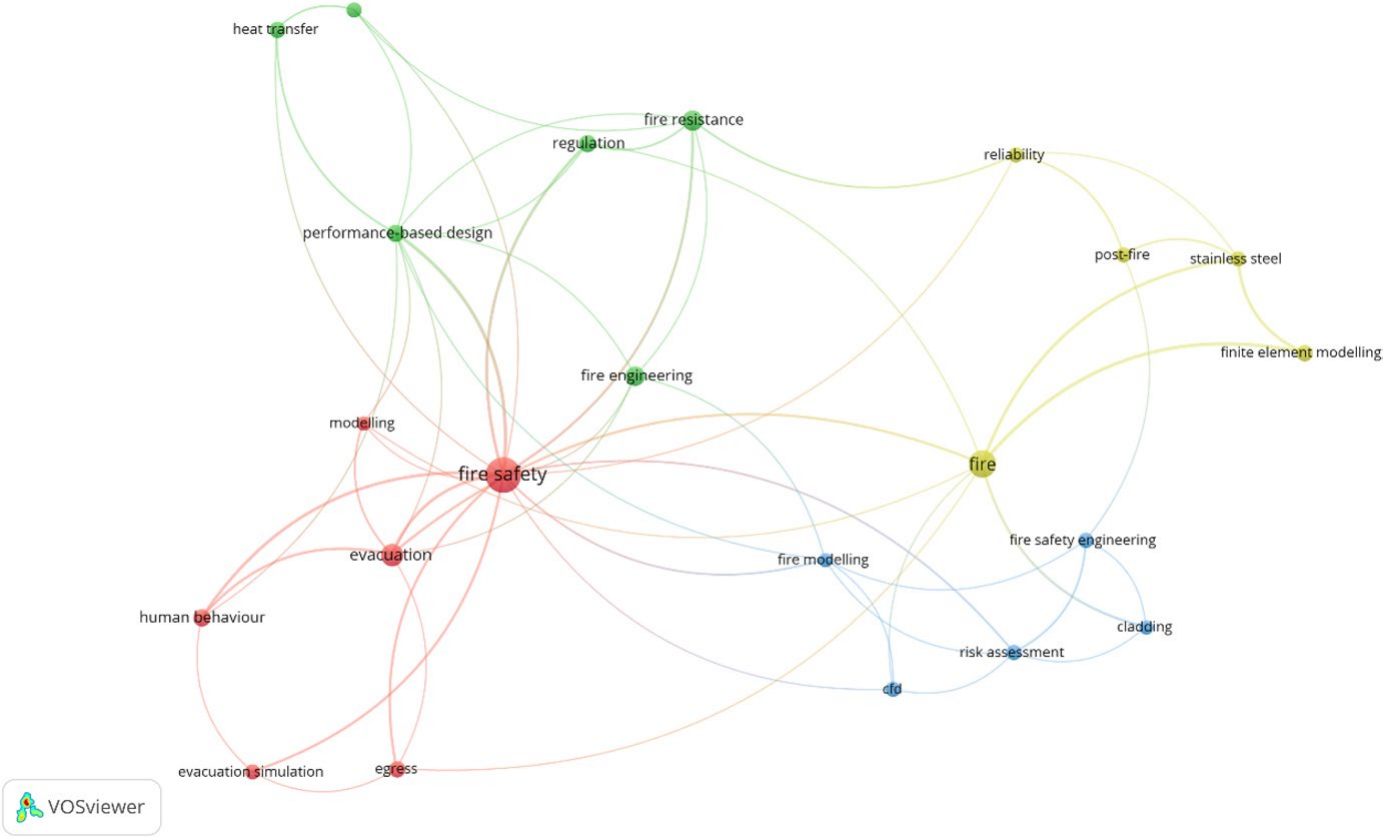
Prominent keywords

Keywords provided by authors are frequently employed to delineate major research domains. In order to identify the thematic focus of the selected articles, a keyword network analysis, which plays a crucial role in identifying the central themes and interconnections within research articles [26] was conducted. In constructing the keyword network, a total of 955 keywords were extracted from the 440 qualified articles and their number of occurrences was counted. A default minimum occurrence threshold of five was established, resulting in the identification of 22 keywords from a total of 955. These were used to construct the keyword network diagram in Fig. 4. Table 4 shows the top 10 keywords in order of their total link strength. The total link strength of these keywords was calculated by the VOSviewer analysis of linkages among the various keywords. The link strength underscores the emphasis and strong connection between fire safety, fire performance-based design, and evacuation of building users, as well as other related keywords.

**Table 4:** Prominent research keywords

S/N	Keyword	Occurrences	Total Link Strength
1	Fire safety	55	36
2	Fire	27	19
3	Evacuation	16	15
4	Performance-based design	8	15
5	Stainless steel	6	11
6	Fire resistance	13	10
7	Finite element modelling	7	9
8	Human behaviour	8	9
9	Regulation	8	8
10	Fire modelling	5	7

### Critical Analysis Findings

The inference from the analysis of the 51 articles extracted from the data collection revealed that there are several practices for ensuring fire safety in buildings. The findings of the review were thematically categorised into four broad approaches, including technological, mitigation, behavioural and regulatory approaches to building fire safety practices. The classification emerged during the analysis through recurring patterns and was guided by existing studies [27]. Table 5 summarises the categorisation of the papers into these distinct approaches and corresponding authors. These categorisations are subsequently discussed in detail.

**Table 5:** Summary of 51 articles extracted and the fire safety approach discussed

Approach	Number of studies	Authors
Technological	20	[4, 14, 21, 28–44]
Mitigation	22	[17, 19, 20, 45–63]
Behavioural	4	[64–67]
Regulatory	5	[68–72]

### Fire Safety Approaches

The analysis of Table 5 shows that building fire safety practices in the UK are mostly approached using technological and mitigation approaches, with 20 and 22 articles, respectively, out of the 51 selected articles discussing these approaches. This can be attributed to the surge in technological advancement and the potential of technological solutions in addressing fire issues [33]. This enables researchers to adopt proactive approaches to mitigates building fire issues, instead of reacting to issues. Meanwhile, limited studies focussed on behavioural approach and regulatory approach to fire safety in buildings. This is alarming as studies like that of Kotzen et al. [19] identified behavioural issues and regulatory concerns as main issues affecting effective building fire safety practices in the UK. There are profound ambiguity and disorganisation in some of the existing recommendations within the regulatory framework related to fire safety in England [19]. These issues result in lack of clarity and difficulty in complying with set regulations and efficient fire safety management in buildings. This is clearly a gap that needs attention from researchers and other stakeholders to provide clarity on how to enhance fire safety behaviour and regulatory compliance to improve fire safety practises in the UK.

#### Technological Approach

A significant strategy for enhancing building fire safety is incorporating cutting-edge technology to improve fire response and risk evaluation. Studies such as [32, 38, 40, 44] have investigated various techniques for evaluating and enhancing fire safety within architectural design, emphasising the adoption of technological approaches to select materials and building elements with improved fire resistance ratings. Other studies have also explore the application of technology or digital solutions to assess the allowable damage and resistance periods of building components in the event of fire [21, 28, 34, 38, 40].

Accordingly, studies have also incorporated information and communications technology (ICT) in improving building fire safety practices. Black et al. [28] investigated the effectiveness of Bluetooth 5.0 in signal transmission under fire conditions, revealing that the technology could sustain signal integrity with minimal degradation, thereby suggesting its utility in bolstering emergency response frameworks. Additionally, Gerges et al. [33] highlighted the significance of smartphones in evacuation scenarios, emphasising their potential to aid individuals in identifying the quickest and safest exit routes. Furthermore, Cowlard et al. [30] developed a framework that enables the utilisation of sensor data to facilitate early detection, enhance building management, and supply essential information to support firefighting efforts. Similarly, Chen et al. [29] utilised computer vision alongside deep generative models to facilitate automated assessments of fire risk within architectural designs. These studies illustrate the capacity of Artificial Intelligence (AI) to significantly improve fire safety practices by offering more efficient and adaptable solutions for fire risk management and mitigation in buildings. The application of machine learning and BIM techniques to facilitate the implementation of frameworks and methodologies for fire safety evaluation and compliance verification has also been identified as a vital technological advancement in fire safety practices. For instance, the framework introduced by Fu [32] employs machine learning techniques to predict failure trends and the likelihood of collapse in steel-framed structures subjected to fire scenarios. This approach enables efficient and precise evaluations of structural failures as a result of fire and, thus enables adequate mitigation strategies to be put in place from the design and construction. Malsane et al. [14] also developed a semantical-based BIM model tailored to Building Regulations for automated compliance checking for fire safety. Similarly, Siddiqui et al. [41] proposed a method for integrating BIM with fire safety engineering analysis, addressing challenges related to data sharing and regulatory compliance. These frameworks and methodologies serve as essential tools for the assessment and assurance of fire safety. Thus, underscoring the continuous development of technological solutions for managing fire hazards and enhancing overall safety standards. These studies underscore the importance of integrating contemporary technology in building fire safety management, emphasising the roles of real-time communication and automated risk assessment in enhancing fire response and prevention methodologies.

Another notable trend in applying technological solutions for fire safety is the advancement and enhancement of fire-resistant materials and structural designs. In their comprehensive parametric study, Suntharalingam et al. [42] examined 3D printed concrete composite (3DPC) wall panels, demonstrating that 3DPC walls combined with Rockwool infilled cavities provide exceptional fire resistance. This indicates that the utilisation of advanced materials can significantly bolster structural integrity in fire scenarios. Also, Kucukler [36] examined the shear resistance and design considerations of stainless steel plate girders subjected to high temperatures and found that the current methodology results in unreliable and inconsistent ultimate strength predictions for these girders during fire exposure. Furthermore, Hopkin & Lay [35] investigated the theoretical debates surrounding the implementation of flappy door systems, presenting both supportive and critical perspectives. The study identifies multiple issues associated with the existing systems, such as the mandatory violations of stair compartmentation, doubts regarding their reliability, the dependence on door closers as a potential single point of failure, the influence of everyday building operations on system efficacy, and the misleading assurance that modelling assessments may offer regarding their sufficiency. Hopkin et al. [4] conducted extensive full-scale fire tests on cross-laminated timber (CLT) enclosures, highlighting the necessity for effective encapsulation techniques to mitigate excessive heat release and improve the fire resistance of timber constructions. These results emphasise that innovative material technologies are currently being explored to enhance the fire performance and resilience of structures.

#### Mitigation Approach

The extensive review of the selected articles uncovered diverse mitigation strategies aimed at enhancing fire safety in buildings. Studies such as [19, 46, 48, 49, 54, 55] discussed the application of risk management strategies in mitigating fire incidents. For example, Inerhunwa et al. [53] and Hansen et al. [48] employed probabilistic risk assessment to analyse the effects of fire service interventions on the structural integrity of fire-resistant materials and stairwells. Showcasing how such approaches can enhance fire safety designs to yield improved outcomes during fire emergencies. Furthermore, Kotzen et al. [19] and [46] analysed existing fire safety regulations related to external living walls, highlighting discrepancies in design and the recommended guidance from fire safety expertise and governance. This allows for the establishment of proper mitigation strategies instead of a reactive approach. Kotzen et al. [19] underscore the need to establish a fire mitigating procedure that accommodates the changing characteristics of building materials and designs, ultimately enhancing the efficacy of fire safety management practices in buildings. Benson & Elsmore [45] stressed a lack of assurance that fire safety expertise is consistently integrated into building and planning processes, suggesting that the existing fire strategies are mostly reactive instead of efficient fire mitigation strategies that contribute significantly to ongoing fire safety challenges. Accordingly, studies such as Cadena et al. [47] and Law et al. [54] have also assessed risks associated with the provision of alternatives and improving resilience for improved fire safety [48, 55]. A compilation of the benefits and challenges of various alternatives has been developed, serving as a resource for fire safety engineers to utilise and implement in their fire risk assessments to develop adequate mitigation strategies for fire in buildings [48]. These studies focusing on integrating probabilistic risk assessment underscore the dynamic and interdisciplinary aspects of fire safety management within the context of risk mitigation. Collectively, these studies demonstrate that addressing risks and uncertainties on time can foster more thorough and effective safety management practices, ultimately mitigating fire risks in intricate and high-risk building settings.

Manes & Rush [59] and Arewa et al. [44] highlight the critical role of thorough document analysis and review techniques in uncovering inconsistencies and gaps in regulations and guidelines that could inform the development of more effective fire mitigation strategies. Manes & Rush [59] utilised quantitative methods to assess how fire brigade response times influence life safety and property protection, demonstrating a clear relationship between these response times and the severity of fire incidents. The study provides valuable insights to enable the optimisation of fire response strategies, the strategic placement of fire stations, and the efficient allocation of resources as part of wider fire safety mitigation strategies. The investigation conducted by [52] highlights how fire mitigation strategies should consider variations in the number of apartments and their occupancy levels as they influence the necessary fire resistance requirements in high-rise residential structures. This is echoed in the work of [47], which advocate for more sophisticated, data-informed methodologies in fire safety design to develop fire mitigation plans that integrate not only structural factors but also occupant behaviour and material performance.

Moreover, the review identifies ongoing deficiencies in fire safety knowledge and its practical application within the building sector. Although the Grenfell tragedy has spurred advancements in fire safety awareness, investigations by [61] and [58] reveal that significant knowledge gaps remain in both educational frameworks and industry practices, particularly concerning fire mitigation strategies and the incorporation of fire safety into building design. The studies also highlight the necessity of considering a range of occupant characteristics—such as disabilities or the presence of pets—when formulating evacuation models [20], indicating that existing evacuation strategies may not adequately address the needs of diverse populations. Findings from [57] further indicate a pressing need for an effective fire mitigation strategy, including more accurate data collection regarding occupant pre-evacuation times, which could enhance the refinement of evacuation models and fire safety engineering methodologies. These investigations highlight the essential requirement for a comprehensive and integrated mitigation strategy for fire safety, one that considers both technical and human elements, in order to foster more resilient and secure built environments.

Other studies [62, 63] provide significant insights into the cost–benefit analysis of fire safety systems to develop effective mitigation strategies and fire safety practices. The research conducted by [53] on the "modified J-value" introduces a quantitative methodology for assessing fire safety initiatives by factoring in costs related to fatalities, injuries, property loss, and the implementation of safety protocols. This methodology highlights the economic ramifications of fire safety choices and also establishes a framework for rationalising investments in fire prevention systems, serving as a crucial resource for mitigation strategies development in fire safety practices. For instance, the investigation by [17] regarding the cost-effectiveness of sprinkler systems in single-family residences challenges traditional assumptions, demonstrating that such systems may not be financially viable unless costs are distributed among multiple units in apartment complexes, thereby fostering more sustainable and economically sound fire protection solutions in urban settings.

#### Behavioural Approach

The findings of this study have revealed the significance of occupant behaviour during fire emergencies and their relationship with fire safety protocols in effective fire safety practices. Templeton et al. [65] examined the influence of trust in fire safety instructions on residents’ reactions during crises in high-rise buildings. Utilising a quantitative online survey with 769 participants, the study, [65] revealed that the clarity of fire safety instructions plays a crucial role in individuals’ readiness to adhere to instructions. The relationship between instruction clarity and individuals’ compliance was found to be mediated by the level of trust in both the content of the instructions and the credibility of their sources. The results emphasise the necessity of providing clear and reliable guidance, as trust enhances compliance and optimises emergency responses, as further substantiated by [73]. Understanding residents’ perceptions and trust in safety information can contribute to improved preparedness and more effective response strategies. Accordingly, [66] emphasised the necessity of taking the general public into account in designing fire evacuation training programs to guarantee both the efficacy and the practical acceptance of such training.

Expanding on the insights regarding residents’ interactions with fire safety protocols, Templeton et al. [64] further explored the dynamics of information sharing and mutual support among residents in high-rise buildings during fire emergencies. Through qualitative analysis derived from 16 focus groups comprising 40 participants, the study indicated that collective self-organisation frequently takes precedence over strict adherence to formal safety protocols. Residents often depend on one another for information and aid, highlighting the vital role of social interactions in crises. The findings suggest that effective fire safety management should incorporate these social dynamics, ensuring that residents are prepared to assist each other, thereby cultivating a more resilient community response in emergencies. These substantiate the unquantifiable impacts of occupants’ behaviour on the effectiveness of any fire safety strategy.

The practical implications of these findings are further highlighted by [63], who investigated occupant interactions with self-closing fire doors using a mixed-methods approach that included both interviews and surveys. Their research revealed that many occupants alter or disable the self-closing mechanisms of fire doors, compromising their effectiveness as a safety feature. This again affirmed the importance of behaviour or attitude over any technology or strategy. As such, it is essential to implement a comprehensive educational initiative to inform occupants about these mechanisms’ critical role and promote adherence to safe practices. This is further stressed in the Fire Safety Regulations (2022) where responsible persons are mandated to carry out regular checks to ensure the functionality of fire safety doors. McDermott et al. [63] suggest that fire safety behaviour can be improved by exploring alternative strategies for continuous user education and active participation in safety protocols.

#### Regulatory Approach

The regulatory aspect of safety management practices emphasises the development and efficacy of building codes and standards in reducing fire hazards. Manes et al. [58] provide an in-depth analysis of resilience in fire safety regulations by delineating its various dimensions and evaluating how UK standards respond to these elements. Their methodology merges theoretical insights with practical implementation, culminating in a fire resilience framework specifically designed for educational facilities. The results highlight the necessity for a systematic approach to resilience, indicating that more precise definitions and guidelines from regulations could greatly enhance safety outcomes.

Similarly, the examination of the UK’s regulatory frameworks has been conducted to identify issues within the existing standards [69, 71, 72]. The evaluation of the frameworks brought to light issues like insufficient heat flux measurement, inconsistent fire loads, and the absence of rigorous controls on debris or molten materials. Additionally, the analysis reveals that these regulatory deficiencies may compromise the effectiveness of façade fire safety strategies [71]. Schulz et al. [70] suggest that the standards do not adequately consider real-world complexities, including the existence of windows and vents, and they criticise the excessive reliance on theoretical studies instead of comprehensive testing. Spinardi et al. [71] contribute to the discourse by indicating that an exclusive reliance on conventional fire resistance standards may inadequately address today’s evolving risks and challenges. Meanwhile Law & Spinardi [68] pointed out that the formulation and implementation of regulations based on ‘codespeak’— the specialised language associated with regulatory frameworks, holds significant influence due to its clarity, certainty, and legal authority. This highlights the need for targeted efforts to holistically revamp the existing regulations to address the fire safety concerns as stressed by Dame Hackitt’s report [75]. Consequently, the inference from the review presented in this study is that policymakers must work together with experts and building users to establish ways of formulating regulations that will incorporate the dynamic nature of fire risks and challenges. This serves to provide flexible regulations specifically targeted at curbing the issues related to fire and ensuring the safety of building users and safeguarding their properties.

### Challenges in Current Fire Safety Practices

**Fig. 5:**
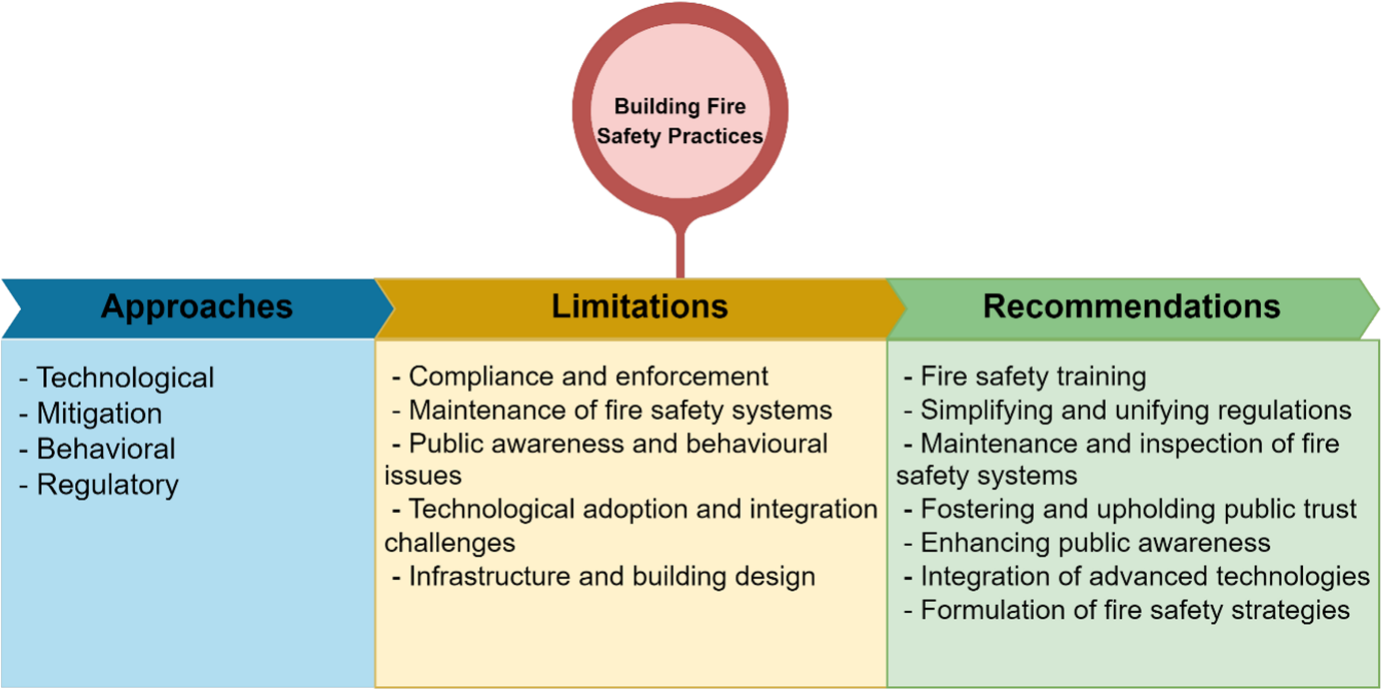
Summary of main review findings for building fire safety practices in the UK

Similar to the thematic classification of building fire safety approaches presented in Table 4, the analysis of several limitations mentioned against each building fire safety approach was thematically categorised. Many of the identified limitations were found to overlap and impact all four approaches, as such, the limitations are discussed collectively rather than in isolation for each approach. Thereafter, recommendations on how to address these limitations to enable effective building fire safety practices are also presented. Figure 5 summarises these limitations and recommendations, which are then discussed in detail in subsequent sub-sections.

#### Compliance and Enforcement Challenges

A prominent challenge facing fire safety practices in the UK is the inconsistent adherence to fire safety regulations and standards [75]. Certain buildings, especially older structures or those located in specific residential areas, often fail to meet contemporary fire safety requirements. This non-compliance is largely due to lack of awareness, financial limitations, inadequate regulatory oversight during their construction or ambiguities in the safety regulations [76]. For instance, buildings constructed before the emergence of modern fire safety standards such as FSO and Approved Document B, do not meet the current regulatory requirements for fire safety [76]. In new buildings, there are ambiguities affecting the enforcement of fire safety regulations [71]. Although inspections are required to overcome such ambiguities, their frequency and thoroughness differ significantly, potentially resulting in lapses in safety standards [75]. This is complicated by the fact that local authorities, often under-resourced, find it challenging to perform regular and comprehensive inspections due to poor accountability and responsibilities in enforcing the standards, leaving certain risks unaddressed [75].

Moreover, the regulatory framework governing fire safety in the UK is intricate, posing several navigational challenges such as partial coverage and ambiguities [70, 71]. Shad et al. [75] claimed that Fire safety regulations are shaped by national and local laws, which can occasionally be disjointed or inconsistent. This lack of cohesion complicates compliance for property owners, particularly those overseeing multiple locations [77]. This complication has always been there, and the tragic event of the Grenfell Tower fire in 2017 prompted intense scrutiny and subsequent reforms in fire safety regulations [75]. These reforms are essential and have led to the implementation of new regulations in the form of the new Building Safety Act, thus ensuring the gradual and consistent compliance with safety standards. However, the challenge still lies in effectively transitioning to these new regulations while addressing the concerns of various stakeholders about bureaucratic obstacles, resource shortages, or resistance from affected parties to enhance effective compliance and enforcement of fire safety regulations.

#### Maintenance of Fire Safety Systems

The effective maintenance of fire safety systems, including alarms, sprinklers, and fire extinguishers, is essential for ensuring their operational reliability during emergencies [78]. Nonetheless, the review presented in this study revealed that various obstacles persist in maintaining them, thus leading to their deterioration. Consequently, the deterioration can result in failures if they are not adequately maintained and upgraded to meet modern standards, an effort that can be expensive and logistically complex [75]. Furthermore, even in more recently built facilities, there are cases where fire safety apparatus is not subjected to regular inspections or maintenance. Fire exits may be obstructed, alarms could be rendered inoperative due to recurrent false activations, and extinguishers might remain unchanged. Such negligence can significantly compromise the efficacy of fire safety protocols.

#### Public Awareness and Behavioural Issues

The role of public awareness and behaviour in fire safety is paramount; however, numerous challenges continue to exist in this area. Although fire safety information is readily accessible, there remains a significant gap in public understanding regarding the critical nature of these safety practices [63, 66]. Some of the reviewed articles have revealed behavioural challenges, such as individuals lacking familiarity with evacuation protocols or not fully grasping the importance of keeping fire doors closed and ensuring that exit routes are unobstructed [64]. These challenges in awareness can result in perilous actions during fire emergencies. Additionally, there is a marked reluctance to engage in fire drills, especially within residential environments. Many individuals perceive these drills as burdensome or superfluous, which contributes to low participation levels [66]. Likewise, there can be a hesitance to adhere to fire safety measures, such as the practice of keeping fire doors closed, which can have dire implications in the event of a fire [66]. In general, this review substantiated the assertion that behavioural challenges impact the effectiveness of fire safety practices.

#### Technological Adoption and Integration Challenges

Technological advancements present considerable opportunities for enhancing fire safety; however, their implementation and integration have been limited because of numerous challenges, including cost. Cutting-edge fire safety technologies, including intelligent detection systems and AI-based fire management solutions, often entail substantial costs [79]. This is evident as some of the solutions require capital-intensive techniques like computer vision and deep generative models [30]. This financial burden can deter many property owners, particularly those in the residential sector or small enterprises, resulting in continued reliance on outdated and less efficient systems.

The intricate nature of modern fire safety technologies, particularly in high-rise structures, poses additional challenges like ensuring and maintaining a working fire system, setting up and developing the capacity of relevant personnel to manage the safety systems, and establishing a sound fire emergency plan to safeguard the systems. [80]. These systems necessitate ongoing updates, maintenance, and specialised technical knowledge for optimal operation. Furthermore, there are apprehensions regarding the reliability of these technologies, especially during power outages or other technical malfunctions in emergencies. Resistance to the adoption of new technologies is also prevalent, particularly within organisations or businesses that have long relied on conventional methods [75]. Such resistance may arise from a lack of awareness regarding the advantages, concerns about financial implications, or uncertainty about how to effectively integrate new technologies with pre-existing systems.

#### Infrastructure and Building Design

The architectural design and structural framework of buildings are integral to ensuring fire safety, yet they present numerous challenges. High-rise structures, in particular, introduce distinct fire safety issues, especially regarding evacuation strategies and the containment of fire spread [80]. The architectural considerations, such as the strategic positioning of fire exits, the efficacy of fire compartmentalisation, and the dependability of sprinkler systems, are essential but frequently insufficient, particularly in older buildings. Upgrading these older buildings to comply with contemporary fire safety regulations can prove quite challenging. This undertaking often entails significant costs, disruption, and technical complexities, especially in heritage-listed properties or those with intricate designs. Property owners may hesitate to pursue such upgrades due to financial implications, perpetuating safety vulnerabilities. Furthermore, the rising urban density in cities exacerbates fire hazards [81]. High population density can hinder evacuation processes and place additional pressure on emergency response teams. In areas with dense populations, the potential for rapid fire spread increases, along with the likelihood of casualties, underscoring the necessity for effective fire safety infrastructure and comprehensive planning.

### Enhancement of Current Fire Safety Practices

Considerable progress has been achieved in improving fire safety measures in the UK. However, there are still deficiencies in regulatory enforcement, public awareness, and the incorporation of emerging technologies. Tackling these issues necessitates a collaborative approach involving policymakers, academic researchers, industry participants, and the general public [82]. By enhancing fire safety protocols through ongoing education, streamlined regulations, and the implementation of cutting-edge technologies, the UK can more effectively protect its structures and inhabitants from the catastrophic effects of fire [82, 83]. This research presents several recommendations to enhance building fire safety in the UK.

#### Fire Safety Training

Fire safety training should be viewed as an ongoing endeavour rather than a singular occurrence. It is imperative to conduct regular drills and refresher courses to ensure that individuals maintain essential knowledge and skills [79]. This will improve the preparedness and reduce risks of fire accidents among participants, foster a heightened state of readiness, thereby minimising the likelihood of panic and disarray during genuine emergencies [66]. The significance of training scenarios that accurately reflect real-life situations cannot be emphasised enough. Training that replicates conditions such as smoke, noise, and restricted visibility equips individuals to better manage the pressures and difficulties associated with an actual fire incident. This indicates that investing in advanced training programs that emulate real-world scenarios is crucial.

#### Simplifying and Unifying Regulations

There is a pressing need to simplify and unify fire safety regulations to enhance their accessibility and ease of implementation [75]. The current complexity and fragmentation of regulations can result in confusion and inconsistent compliance [70, 71]. By streamlining these regulations, adherence can be improved, thereby reducing the risk of oversight. Additionally, it is vital to provide timely updates to fire safety regulations in light of new research, emerging threats, and technological progress [76]. The Grenfell Tower fire serves as a stark reminder of the perils associated with outdated regulations, highlighting the necessity for a flexible regulatory framework that adapts to evolving circumstances [75].

#### Maintenance and Inspection of Fire Safety Systems

The regular upkeep of fire alarms, sprinklers, and other safety apparatus is vital to guarantee their proper operation in times of need [80]. This underscores the dangers linked to the failure to conduct routine inspections and maintenance, which may result in equipment malfunction during critical situations. The issue of ageing infrastructure in the UK necessitates prompt upgrades to obsolete fire safety systems [76]. Retrofitting older structures to comply with contemporary standards is not merely a legal obligation but also a moral responsibility aimed at safeguarding lives [76]. This highlights the necessity of prioritising financial resources and support for such enhancements, especially in buildings deemed high-risk.

#### Fostering and Upholding Public Trust

The efficacy of fire safety protocols is significantly influenced by the public’s confidence in authorities and the information they disseminate. Communication during emergencies must remain clear, consistent, and trustworthy [64]. This accentuates the importance of sustained efforts to foster and uphold public trust, which includes transparent dialogue and regular interaction with communities. Additionally, the significance of straightforward and succinct communication during fire emergencies cannot be overstated. The success of emergency responses may be jeopardised by ambiguous or contradictory messages, potentially leading to hazardous actions [65]. Consequently, it is imperative to establish and routinely practice clear communication protocols to ensure that all involved parties comprehend their responsibilities and the information being shared.

#### Enhancing Public Awareness

The significance of robust public education initiatives aimed at enhancing awareness of fire safety and promoting constructive behaviours is paramount [63, 66]. Such initiatives must be ongoing and flexible to effectively engage various demographics, particularly those vulnerable populations that may face increased risks in the event of a fire [66]. Promoting adherence to fire safety protocols often necessitates more than mere educational efforts; it may also require the introduction of incentives. Insights from behavioural economics indicate that offering concrete incentives for upholding fire safety measures, such as lower insurance premiums or tax deductions, can significantly enhance compliance and involvement in safety initiatives.

#### Integration of Advanced Technologies

Furthermore, the integration of advanced technologies, including intelligent fire detection systems and AI-based evacuation strategies, has the potential to substantially improve fire safety results. Nevertheless, these technologies must be seamlessly incorporated into existing frameworks and supported by human oversight [28, 30]. Technology should be regarded as an enhancement to traditional safety measures rather than a replacement. Additionally, it is essential to tackle the obstacles that hinder the adoption of new technologies, such as financial constraints, complexity, and resistance to change. Addressing these challenges necessitates focused strategies, including the provision of financial incentives, the implementation of training programs, and the demonstration of the practical advantages of these technologies to relevant stakeholders.

#### Formulation of Fire Safety Strategies

Finally, formulating fire safety strategies that are tailored to the specific contexts of various environments, including high-rise structures, educational institutions, healthcare facilities, and residential areas, is necessary [80]. A uniform approach is frequently insufficient; therefore, safety protocols must be customised to address the distinct risks and requirements inherent to each environment. Additionally, there is a pressing need for flexibility and adaptability within fire safety practices. Fire safety plans should be dynamic, permitting modifications in response to emerging information, alterations in building usage, or the integration of new technologies [80]. This level of adaptability is essential for effectively addressing the diverse and changing landscape of fire risks in the UK.

## Conclusion

Several studies have directed efforts towards ensuring building fire safety practises in the UK to improve the regulatory guidance and optimise the practise. However, there are dearth of studies that have provide comprehensive analysis of current building fire safety practices to support their improvement. Consequently, this study presents a systematic review of extant literature pertinent to building fire safety practices in the UK, with the aim of identifying the current safety approaches, challenges facing these approaches and then recommendation to improve them. The study conducted both bibliometric and critical analyses of articles related to building fire safety in the UK. The bibliometric analysis conducted on all qualified articles from 1962 revealed an increase in publications from 2004. Additionally, the analysis found Fire Safety Journal, Fire Risk Management, Fire Prevention and Fire Engineers Journals, and Fire Technology to be the top four outlets for disseminating building fire safety research outputs. Furthermore, the analysis uncovered the leading authors for building fire safety to be Shields, T.J., Silcock, G.W.H., and Boyce, K.E. Finally, the bibliometric analysis found Fire safety, Fire, and Evacuation to be the most occurring keywords.

The critical analysis of 51 qualified articles selected from 2010 classifies fire safety approaches using thematic analysis into four distinct categories: technological, mitigation, behavioural, and regulatory, each playing a vital role in improving fire safety in buildings. The study further identified several challenges facing current practices in building fire safety. These include issues related to regulatory compliance and enforcement, maintenance of fire safety systems, public awareness and behavioural issues, technological adoption and integration challenges, and infrastructure and building design challenges. To address these identified issues, this study provided recommendations for improving building fire safety through fire safety training, simplifying and unifying regulations, maintenance and inspection of fire safety systems, fostering and upholding public trust, enhancing public awareness, integrating advanced technologies, and formulating fire safety strategies.

This study contributes to the existing literature by emphasising the interdisciplinary nature of fire safety management, demonstrating how technological, regulatory, and behavioural dimensions interacts with fire mitigation in building. It proposes for an integrated framework that harmonises these various components to effectively enhance building fire safety outcomes. However, the review’s focus on the UK may limit the applicability of its findings to other regions. Conducting comparative studies across different nations could yield valuable insights into best practices and identify potential enhancements to the UK’s fire safety regulations. Furthermore, it is advisable to explore the sociocultural dynamics that influence building fire safety compliance to inform the development of evidence-based building fire safety frameworks.

## Data Availability

The authors declare that the data supporting the findings of this study are available within the paper using the DOI presented in the reference section. Full details of the search terms and the sources were provided in the methodology section.
